# Micronutrient Status and Dietary Diversity of Women of Reproductive Age in Rural Pakistan

**DOI:** 10.3390/nu12113407

**Published:** 2020-11-06

**Authors:** Anna K. M. Brazier, Nicola M. Lowe, Mukhtiar Zaman, Babar Shahzad, Heather Ohly, Harry J. McArdle, Ubaid Ullah, Martin R. Broadley, Elizabeth H. Bailey, Scott D. Young, Svetlana Tishkovskaya, Muhammad Jaffar Khan

**Affiliations:** 1 Lancashire Research Centre for Global Development, Faculty of Health and Wellbeing, University of Central Lancashire, Preston PR1 2HE, UK; AKMBrazier1@uclan.ac.uk (A.K.M.B.); HOhly1@uclan.ac.uk (H.O.); 2 Department of Pulmonology, Rehman Medical Institute, Peshawar 25000, Pakistan; mza38@hotmail.com; 3 Institute of Basic Medical sciences, Khyber Medical University, Peshawar 25100, Pakistan; babar.kmu@gmail.com (B.S.); drubaid.ullah@yahoo.com (U.U.); jaffar.khan@kmu.edu.pk (M.J.K.); 4 School of Biosciences, University of Nottingham, Sutton Bonington Campus, Leicestershire NG7 2RD, UK; h.mcardle@abdn.ac.uk (H.J.M.); martin.broadley@nottingham.ac.uk (M.R.B.); liz.bailey@nottingham.ac.uk (E.H.B.); Scott.Young@nottingham.ac.uk (S.D.Y.); 5 Lancashire Clinical Trials Unit, Faculty of Health and Wellbeing, University of Central Lancashire, Preston PR1 2HE, UK; STishkovskaya@uclan.ac.uk

**Keywords:** zinc, iron, selenium, micronutrients, malnutrition, dietary diversity, Pakistan, women of reproductive age (WRA)

## Abstract

Consuming a diverse diet is essential to ensure an adequate intake of micronutrients. The aim of this study was to assess the nutritional status and dietary diversity of women of reproductive age (WRA) living in a marginalized community in rural Pakistan. Forty-seven WRA (35 ± 7 years old) who were not pregnant or lactating at enrollment, were recruited to participate in the study. Twenty-four-hour dietary recall interviews were conducted by the study nutritionist, and the data collected were used to create a minimum dietary diversity for women score (MDD-W) on five occasions during the monsoon and winter seasons (October to February). Nutritional status was assessed using anthropometry and biochemical markers of micronutrient status. Height and weight were used to determine body mass index (BMI), and mid-upper-arm circumference was measured. Plasma zinc, iron, and selenium concentrations were measured using inductively coupled mass spectrometry, and iron status was assessed using serum ferritin and blood hemoglobin concentrations. The mean (±SD) food group diversity score was 4 ± 1 with between 26% and 41% of participants achieving an MDD-W of 5. BMI was 27.2 ± 5.5 kg/m^2^ with 28% obese, 34% overweight, and 6% underweight. The prevalence of zinc deficiency, based on plasma zinc concentration, was 29.8%; 17% of the participants had low plasma selenium levels; 8.5% were iron deficient; and 2% were suffering from iron deficiency anemia. The findings indicate that the women living in this community consume a diet that has a low diversity, consistent with a diet low in micronutrients, and that zinc deficiency is prevalent. Public health interventions aimed at increasing the dietary diversity of WRA are needed to improve the micronutrient intake, particularly of zinc, in this population.

## 1. Introduction

Malnutrition affects every country in the world [1] and refers to both undernutrition and overnutrition. Undernutrition in adults is characterized by wasting and underweight. Overnutrition is manifested as overweight, obesity, and diet-related non-communicable diseases such as diabetes, stroke, and heart disease [2]. The United Nations Decade for Nutrition Action was launched in 2016, as a commitment by member states to focus efforts on reducing malnutrition in all its forms, leaving no-one behind through bringing together policy, investments, and programs to improve access to nutritious and sustainable diets for all [3]. Despite this, suboptimal micronutrient status in women of reproductive age (WRA) remains a challenge. Moreover, recent reports indicate a concerning global trend toward an increase in overweight and obesity, often occurring simultaneously with undernutrition in low- and middle-income countries (LMICs) [4]. This so-called double burden of malnutrition occurs within populations, communities, and families where access to affordable, nutritious food is limited, and the emphasis is placed on purchasing low-cost, energy-rich staples, such as rice and grains, that are low in micronutrients [4]. This may result in multiple micronutrient deficiencies, particularly of iron, zinc, vitamin A, and iodine [5,6]. Selenium deficiency may also be prevalent in areas where the soil selenium content is low or unavailable for uptake by crops due to the soil type [7]. This combination of increased obesity and increased undernutrition in the same population, together with micronutrient deficiency, is often referred to as the triple burden of malnutrition [8]. Dietary micronutrient intake can be improved by broadening the range of foods consumed that are affordable and locally available and is an important part of the strategy for alleviating malnutrition, alongside supplementation, fortification, and biofortification [9].

Pakistan is affected by the triple burden of malnutrition. According to the most recent Pakistan National Nutrition Survey (PNNS) conducted in 2018 [10], 14% of WRA were undernourished, which is an improvement from 18% recorded in the previous PNNS of 2011 [11]. However, overweight and obesity have risen from 19.4% and 9.5%, respectively, in 2011 to 24.0% and 13.8%, respectively, in 2019. This trend is observed in both rural and urban communities [10,11]. This increase in the prevalence of overnutrition is likely to be linked with household food insecurity, where rapid changes in the food systems have led to the increased consumption of highly processed foods and sugary beverages, which are available at a much lower cost than a nutritious diet [12]. The PNNS 2018 revealed that over a third of households are food insecure, with 18.3% falling into the severe food insecurity category [10].

Vitamin A deficiency affects 27.3% of WRA, with a higher prevalence in rural settings compared with that in urban communities. At a national level, the prevalence of iron deficiency anemia (IDA) is 18.2%, with up to a quarter of women reported to have IDA in Balochistan Province. Zinc deficiency is more common in rural settings (24.3%) than in urban (18.7%) communities, and iodine deficiency affects 17.5% of WRA, despite a national universal salt iodization policy implemented in 1994 [13].

To date, studies on dietary diversity in Pakistan have focused on infants and children [13,14,15] and pregnant women [16,17,18], as adequate nutrition from gestation to the first 24 months of life is critical for physical and cognitive development [19]. The nutritional status of the mother has a direct bearing on the long-term health status of the infant [20], and poor nutritional status can result in restrictive growth leading to low birth weight, increasing the risk of childhood infections and mortality, as well as increasing the risk of stunting, wasting, and impaired cognitive development in later life, if not addressed [20]. However, there is a lack of dietary diversity data on Pakistani WRA, who are not pregnant nor lactating.

The aim of this study was to assess the dietary diversity and nutritional status of WRA, living in a low-resource community in rural Pakistan, with a focus on zinc, iron, and selenium. This study forms part of a larger research program to explore the effectiveness of wheat biofortification as a strategy to improve the micronutrient density of a staple crop in Pakistan (known as the BiZiFED study) [21]. Understanding the diversity of the typically consumed diet and prevalence of key micronutrient deficiencies in the target community was an important first stage in this program.

## 2. Materials and Methods

### 2.1. Context and Participant Recruitment

The study was undertaken in a rural community living in the brick kilns close to Peshawar in Khyber Pakhtunkhwa (KPK) Province, Pakistan. The community are involved as part of a larger 18-week cross-over randomized control trial (RCT), BiZiFED, that was conducted during the months of October 2017 to February 2018. The full protocol has been published [21] and registered with the ISRCTN registry (study ID ISRCTN83678069). Ethical approval was granted from the lead institution, the University of Central Lancashire (reference number: STEMH 697 FR). The data presented in this paper are a descriptive, cross-sectional time-series analysis of the dietary intake data collected at 5 time points during the 18 weeks study and blood biochemical data collected during the baseline period (October 2017).

The target community is comprised of approximately 5000 households, made up of 10 villages (clusters), which are served by a single health center. The target was to recruit 50 households to be able to accommodate a 20% attrition rate based on the primary outcome measure of the RCT [21]. Out of the 10 clusters, 5 were randomly selected. Ten households from each cluster were then randomly selected using the lottery method for invitation to participate in the study. The head of the household was approached, the purpose of the study was explained and, if eligible, the family was invited to participate in the trial. Inclusion criteria were that the household included a female aged 16–49 years who was neither pregnant nor breastfeeding and not currently consuming nutritional supplements. When there was more than one eligible woman in the household, one was selected with agreement from the head of the household. There were no additional exclusion criteria.

If the head of the household declined, another house from the cluster was randomly selected, and the invitation process was repeated. If the head of the household agreed, then the eligible females within the household were approached, and the purpose of the study was explained. Literacy rates in this community, particularly amongst women are low [22], therefore the study information and consent forms were translated from English into the local language (Urdu) and explained verbally. Consent from the participating female was indicated by signing with initials or an X on the consent form. As has been previously discussed, community acceptance is critical in these studies [21], hence the recruitment process was closely overseen by the project manager, because he was known to the community through previous health-related research projects in which the Abaseen Foundation Pakistan (AFPK) has been the implementation partner.

### 2.2. Data Collection Procedure

Participant demographics were collected at baseline (T1). Anthropometric measurements, including height, weight, mid-upper-arm circumference (MUAC), along with blood samples for biochemical analysis of nutrition and general health status were collected at baseline, and at four additional timepoints (T2–T5), at intervals of 4 weeks during the intervention period. At these same timepoints, dietary diversity was determined based on 24 h dietary recall data. In this manuscript, we are reporting the dietary diversity data collected across all five timepoints, and the socioeconomic, demographic, anthropometric, and biochemical data collected at baseline. This is to establish the typical local diet and the potential consequences for nutritional status in this low-resource setting. Full longitudinal dietary analysis and biochemical status data resulting from the intervention will be reported separately.

### 2.3. Demographics and Socioeconomic Status

Information regarding demographics and socioeconomic status was collected at baseline using an interviewer-administered questionnaire. This included participant age, education level achieved, and general health information. Household information included the number of family members and the number of rooms in the house, house ownership, structure material, water source, kitchen and toilet facilities, and household income.

### 2.4. Anthropometry

The participant height, weight, and MUAC were measured as previously described [21]. In brief, prior to weight-taking heavy clothing (like burqa) was removed. The participant was then asked to stand on the scale (model EB9064, CAMRY, Kowloon, Hong Kong) and relax, with their arms at their side, feet were positioned close together with their weight evenly distributed on both feet. Height was measured by a stadiometer. Before height was measured, footwear and socks were removed. Then the participants were asked to stand with heels placed together; back of the heels, buttocks, and shoulder blades touching the back plate/stick, and the head was positioned in the Frankfurt horizontal plane. MUAC was measured with a non-stretchable MUAC measuring tape at a point equidistant between the acromion process of the left scapula and the olecranon process of the left ulna. BMI was calculated using the standard formula: weight in kg/(height in m)^2^. The nutritional status of the participants was evaluated using BMI and MUAC, with cutoff points of <18.5 kg/m^2^ and <22 cm used, respectively, to identify adult undernutrition [23,24].

### 2.5. Dietary Diversity

The minimum dietary diversity for women score (MDD-W) was utilized to assess the diversity of the participants’ diets. A score of 5 is required to indicate micronutrient adequacy of the diet at a population level [25]. The MDD-W and food group diversity score were calculated based on ten food groups: 1. grains, white roots and tubers, and plantains; 2. pulses (beans, peas and lentils); 3. nuts and seeds; 4. dairy; 5. meat, poultry and fish; 6. eggs; 7. dark green leafy vegetables; 8. other vitamin A-rich fruits and vegetables; 9. other vegetables; 10. other fruits. Food group consumption was recorded by entering “1” if the food group was consumed and “0” if it was not. A minimum quantity of 15 g was required for a food group to be recorded. A food group diversity score was calculated out of ten. Other food categories included in the analysis were as follows: “other oils and fats”, “sugar-sweetened beverages”, “condiments and seasonings”, “other beverages and foods”, and “sweets”. These categories are not included in the MDD-W due to the negligible contribution they make to the micronutrient intake. However, “other oils and fats” was analyzed separately as they provide a significant contribution to the participant’s energy intake. Additionally, “sugar-sweetened beverages” and “other beverages and foods” were analyzed separately, as there are compounds in some beverages that may have an inhibitory effect on micronutrient absorption; these categories were analyzed and presented separately to the MDD-W and food group diversity score [25].

The multiple pass method was used to collect quantitative diet data [26]. The participants were asked what foods and beverages were consumed during the 24 h period from when they awoke, and household measures were used to determine portion size. A series of questions were asked to determine hidden foods, such as sugar in tea, and to gather recipe details for composite dishes [26]. Interviews were conducted by the study nutritionist at five time points throughout the 18 week study. There were no special occasions during the project that would elicit abnormal eating patterns (e.g., Ramadan).

### 2.6. Blood Biochemistry

Whole blood (non-fasting) was drawn from the antecubital vein of the study participants through a butterfly needle into three plastic vacutainers (BD Diagnostics, Switzerland) as follows. Blood (2 mL) was collected into a tube containing Ethylenediaminetetraacetic acid (EDTA) anticoagulant for complete blood count (CBC), hematocrit, hemoglobin (Hb), and mean corpuscular volume (MCV). These were measured on whole blood using an automated hematology analyzer (Sysmex XP-100, 19 Jalan Tukang, Singapore).

For plasma trace mineral analysis, whole blood (5 mL) was collected into trace-element-free tubes containing EDTA anticoagulant. The plasma was separated by centrifugation within 40 min, and aliquots (300 µL) were stored at −80 °C. Plasma samples were shipped on dry ice to the University of Nottingham for mineral concentration analysis. Elemental concentrations of zinc and selenium in plasma samples were determined using inductively coupled plasma–mass spectrometry (ICP-MS; Thermo Fisher Scientific iCAPQ, Thermo Fisher Scientific, Bremen, Germany). Full details of instrument conditions and quality control data are provided in Appendix A.

For serum ferritin (SF) and soluble transferrin receptor (STfR) analysis, blood (5 mL) was collected into a tube containing a silica clot activator and allowed to stand on ice for 30 min. The serum was separated by centrifugation, and aliquots (300 µL) were stored at −80 °C. Serum ferritin (SF) and soluble transferrin receptor (STfR) were measured using an enzyme-linked immunosorbent assay (ELISA) (Ferritin ELISA: Chemux Bioscience, Inc., Hayward, CA, USA; STfR ELISA: Cusabio, Houston, TX, USA).

Iron deficiency was determined by serum ferritin levels below 15 μg/L [27], anemia by Hb levels below 120 g/L [28], and IDA was determined by the presence of both iron deficiency and anemia [29]. Zinc deficiency was defined as a plasma zinc concentration (non-fasting) (PZC) of <660 µg/L [30]. Low serum selenium levels were defined as <80 µg/L, which is the concentration required for the optimal production of selenoproteins and glutathione peroxidases [31].

### 2.7. Statistical Analyses

Descriptive statistics are presented for the participants’ characteristic MDD-W score, anthropometry, and blood biochemistry. These were calculated using SPSS version 27.

## 3. Results

### 3.1. Participant Characteristics

The participant demographic and baseline anthropometric data are summarized in Table 1. Of the 50 women recruited, 47 completed the baseline (time point 1) data collection, and 45 women completed the 18 week study. The reasons for drop-out were family bereavement, migration out of the community, and illness. One further participant withdrew at timepoint 4 due to refusal to give a blood sample, and one withdrew at timepoint 5 due to illness. The overall participant retention was 90%. The average age of the participating women at baseline (n = 47) was 35 ± 7 years, with a range of 22–48 years. All of the women were married; one had completed education up to the age of 16 years; 98% were illiterate. None of the women were taking oral contraceptives, and only one woman had taken any medication in the previous month (antibiotic).

The women lived in extended family households, with between 1 and 4 adult women, 1–3 adult men, and an average of 4 children. The majority of the homes were built of mud and straw, known as katcha (66%) and comprised on average 2 rooms. Most households had a designated indoor kitchen area (85%), but some cooked outside in an open space (13%). The source of drinking water was a local bore hole (79%), and most had a toilet facility within the household compound (81%). In all, 85% of the respondents reporting being the homeowner, with 9% renting.

### 3.2. Anthropometric Measurements

The mean ± SD BMI at baseline was 27.1 ± 5.6 kg/m^2^. More than half of the women were either overweight (34%) or obese (28%), with 3 women (6%) falling into the underweight category [24]. One woman fell below the MUAC cutoff value of 22.0 cm indicating undernourishment [23].

### 3.3. Dietary Diversity

The MDD-W used was comprised of 10 food categories with 5 indicating minimum dietary diversity, and a food group diversity score was calculated with a range of 0–10 [25]. All foods consumed by the participants in each of the categories are shown in Table 2.

**Figure 1:**
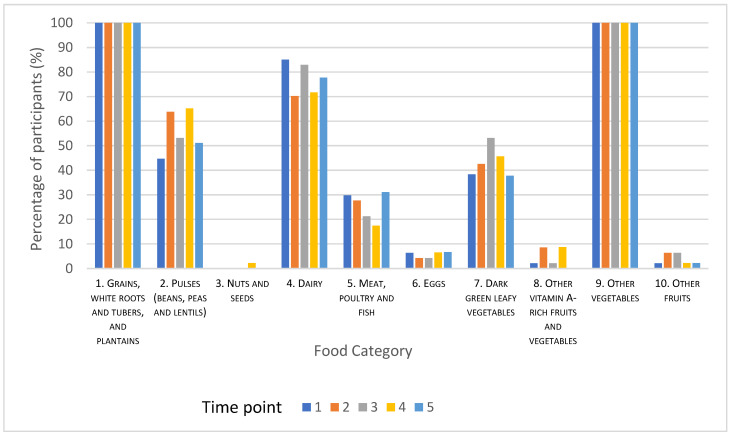
The percentage of participants that consumed at least one food from each category over the five study time points.

The frequency of consumption for each of the 10 food group categories is illustrated in Figure 1. All the participants ate “grains, white roots and tubers, and plantains” and “other vegetables” at all time points. “Dairy” was consumed by between 70% and 85% of participants across the five time points. Between 45% and 65% and 38–53% of the participants consumed “pulses (beans, peas, and lentils)” and “dark green leafy vegetables”, respectively. “Meat, poultry, and fish” was consumed by 17–30% of the participants. The least frequently consumed food groups were “nuts and seeds”, “eggs”, “vitamin A-rich fruit and vegetables”, and “other fruits”, which were consumed by <10% of the participants at each time point.

The mean (±SD) number of food groups consumed was calculated for each time point. Scores ranged from 2 to 7 (out of a possible 10), with a mean of 4 ± 1 at each of the five times points. The frequency distribution of the scores at each time point are shown in Table 3 and shows that between 26% and 41% of women achieved MDD-W at all 5 time points.

The mean (±SD) consumption for the categories not included in MDD-W was “other oils and fats” 100% ± 0%, “sugar-sweetened beverages” 97% ± 4%, “condiments and seasonings” 100% ± 0%, “other beverages and foods” 6% ± 4%, and “sweets” 2% ± 2%.

### 3.4. Blood Biochemistry

The mean ± SD, median, and lowest and highest values for PZC, iron, and selenium concentrations and hematological parameters found in our study group are presented in Table 4, along with the reference ranges and/or cutoff values, and number of participants who fell below the reference range or cutoff value.

A third of the participants had a plasma zinc concentration (PZC) below the cutoff value of 660 µg/L (non-fasting) [30]. Low plasma selenium and iron concentrations were present in 17% and 2% of participants, respectively.

The WHO defines iron deficiency as a serum ferritin level below 15 μg/L, anemia as a Hb level below 120 g/L, and IDA as the presence of both iron deficiency and anemia [29]. Based on this definition, only 1 participant had IDA. None of the participants had elevated soluble transferrin receptor protein concentrations.

## 4. Discussion

This study was conducted in a low-resource community in rural Pakistan where female adult literacy levels are very low. Water is collected from a bore hole or tube well that serves a cluster of households and stored in large containers within the household compound. The anthropometric data reveal that over half of the participants are overweight or obese. In addition, the majority of the MDD-W scores for the women participating in this study, measured at 5 timepoints, fall below the minimum score of 5, indicating low dietary diversity. Analysis of the blood samples revealed that, based on PZC, almost a third of the participants are zinc deficient, and 17% have low plasma selenium concentrations; however, surprisingly, iron deficiency does not appear to be a major problem in WRA in this community.

The PNNS 2018 [10] reports that the overall prevalence of overweight and obesity among WRA is 24% and 13.8%, respectively, with a slightly lower prevalence of both in rural compared with urban communities. The PNNS data also indicate a higher prevalence of overweight and obesity in Khyber Pakhtunkhwa (KP) than the national average, at 28.2% and 15.0% [10]. The data from our study reveal a similar prevalence of overweight (34%) but a higher prevalence of obesity (28%) among the study participants.

To our knowledge, this is the first time the MDD-W has been used in non-pregnant women in Pakistan. The MDD-W scores fell below 5 for the majority of the women at all five time points, which suggests that micronutrient deficiencies are likely in this population. The MDD-W is a dichotomous indictor of whether or not women 15–49 years of age have consumed at least five out of ten defined food groups the previous day or night, validated at the population level, in low-resource settings [25]. A food group diversity score ranging from 0 to 10 was developed to further analyze the percentage of WRA consuming above or below the MDD-W of 5 [25]. Research from a rural community in the Sindh province found only 20% of pregnant women reached minimum dietary diversity [17], this is lower than in the present study and suggests pregnant women from the Sindh province live in a poorer and more restricted environment, with less access to a wide variety of nutritious foods than the current study population, though further research would be required to confirm this. The percentage of women reaching minimum diversity was lower than in previous research conducted on pregnant women in Islamabad, where 89% achieved minimum diversity [16]. However, this is not unexpected as the women in Islamabad were better educated [16] and would likely have access to a wider variety of foods in the markets and shops of an urban environment. Nevertheless, despite the high percentage of pregnant women consuming minimum dietary diversity, there was a much higher prevalence of anemia (29.1%) [16] compared to that in the present study.

According to the recent PNNS, IDA affects 18.7% of WRA living in rural areas of Pakistan, with a slightly lower prevalence in urban regions (17.4%) [10]. Our study revealed that only 1 participant (2%) had IDA, 13% were anemic based on Hb, and 8.5% had low iron stores based on serum ferritin levels. This is also in contrast to previous research conducted at the provincial level in KPK where the prevalence of anemia was found to be 51% [35].

This low prevalence of IDA was an unexpected finding, because the diet was low in readily bioavailable heme-iron found in animal products, with a maximum of a third of the participants consuming “meat, poultry, and fish” at each time point. It is also hard to explain alongside the relatively high prevalence of zinc deficiency in this community (29.8%), which is higher than the national average for rural settings (24.3%) and the provincial prevalence of zinc deficiency (15.9%) [10]. Deficiencies of both zinc and iron were expected as the main staple of the diet was wheat, in the form of breads (naan, chapatti, and paratha) consumed by all the participants on a daily basis. Wheat is high in phytic acid, which inhibits zinc and iron absorption; zinc and iron absorption depends on the solubility of the zinc complex and the form of iron consumed (heme or non-heme) [36]. Phytic acid (PA) chelates with both iron and zinc from dietary sources and zinc from endogenous sources, forming an insoluble complex, rendering the zinc and iron unavailable for absorption [36,37]. Tea, either green or black, with or without sugar, was also consumed by all the participants. Tannins, a component of green and black tea, have been shown to increase the inhibitory effect of PA, by forming insoluble complexes with the PA, zinc, and iron, thus further reducing the absorption of zinc and iron in the gastrointestinal tract and contributing to their deficiencies [38,39]. Tannins are also present in vegetables and legumes, which could also marginally contribute to the inhibitory effect of PA, though tannins from tea have been shown to have a greater inhibitory effect than tannins from vegetables and legumes [36,39]. It is possible that the use of an iron griddle and/or iron-based cooking pots could have been a source of iron into the diet [40]. Iron has been shown to leach from cooking equipment to a varying degree, depending on the acidity of the food and the type of cooking equipment used (cast iron pot, stainless steel, or iron ingot) [40,41,42]. Research conducted in Malawi showed a significant increase in Hb levels in adults when an iron cooking pot was used continuously for 6 weeks [40]. However, this phenomenon has yet to be explored in Pakistan and warrants further investigation. Analysis of water from this community did not indicate a high level of iron in the ground water (unpublished data).

The PNNS of 2018 or 2011 did not measure biomarkers of selenium status [10,11]. Our study revealed that 17% of participants had below-optimal plasma selenium concentrations and that the mean plasma selenium concentration was 96.1 ± 17.1 µg/L. This is lower than the serum selenium concentrations reported from healthy adult females in Rawalpindi and Islamabad (239.92 ± 138 μg/L) [43]. The discrepancy could be explained by spatial variation in grain selenium in Pakistan, since selenium levels in plants vary accoording to the soil selenium concentrations [7].

A strength of this study was that the MDD-W score was calculated on five different occasions, over an 18 week period, providing an overview of dietary diversity among WRA in this community over time. The study was conducted between October and March, which is the wet monsoon and cool winter season in Pakistan. We would not expect to see any major seasonal variations in the availability of typically consumed foods during this period, and this is reflected in the absence of any trends in the foods consumed across the five time points illustrated in Figure 1. It may have been expected that the provision of flour to the households may have altered the typical diet by either displacing other, perhaps, more nutrient-dense foods or releasing a portion of the household income usually spent on flour to be used to buy additional foods. The amount of flour provided to each household was enough only to meet the household needs for the baking of bread (paratha, chapatti, and roti), which is consumed with every meal. The replacement was therefore like for like, replacing household purchased flour with study-supplied flour. It is possible that additional, nutrient-dense food was purchased with the funds released into the household budget. The data presented here may therefore be an overestimation of the diversity of the diet in this community. A limitation of this study is the relatively small sample size, which may have implications in terms of the representability and generalizability of the findings to the community. Although the focus of the study was (WRA), households are comprised of 2 or 3 generations of extended family members, typically up to 9 individuals. Meals are prepared for all the household members together, and although portion sizes may vary, we are confident that the diversity score is representative of the diet of all household members.

In summary, this analysis of dietary diversity and nutritional status of WRA living in a low-resource community in NW Pakistan reveals that the typical diet has a low diversity, likely to result in micronutrient deficiencies. This was confirmed by a low selenium and PZC in this group of women, with 30% of the women falling below the threshold PZC sufficiency. The International Zinc Nutrition Consultative Group recommends that when the prevalence of low PZC in the population is >20%, the risk of zinc deficiency is considered elevated and should be addressed through public health nutrition interventions [30]. One such intervention for this community is advice on how to improve dietary diversity using inexpensive foods that are locally available. The authors are conducting a follow-up study which includes a Cost of the Diet analysis that will survey the markets commonly used by this community, to develop food-based recommendations aimed at improving the community’s dietary diversity and micronutrient intake, based on the most nutritious foods available at the lowest cost [44].

## Figures and Tables

**Table 1:** Participant characteristics (n = 47).

Characteristic	Mean (SD)	N (%)	Median	Range
Demographic features				
Age (in years)	36 (7)		35	22–48
Education level				
None		46 (98)		
Matriculation *		1 (2)		
Married		47 (100)		
Anthropometric Features				
Height (m)	1.56 (0.06)		1.56	1.47–1.70
Weight (kg)	66.3 (14.2)		67	44–100
BMI ^ƾ^ (kg/m^2^)	27.1 (5.6)		27.0	17.4–41.4
Underweight (<18.5)		3 (6)		
Healthy weight (18.5–24.9)		15 (32)		
Overweight (25–29.9)		15 (34)		
Obese (>30)		13 (28)		
Mid-upper-arm circumference (cm)	29.0 (4.3)		29	21.5–39.0
Household features				
No. adult women in the household	2.0 (0.8)		2	1–4
No. adult men in the household	1.8 (0.7)		2	1–3
No. children in household (total)	4.5 (1.8)		4	1–8
Number of rooms in the house	2.4 (1.3)		2	1–6
Structure of House				
Bricks		16 (34)		
Katcha ^¶^		31 (66)		
Toilet facility present				
Yes		38 (81)		
No		9 (19)		
House ownership				
Own		40 (85)		
Rent		4 (9)		
Free tenant ^ǂ^		3 (6)		
Food, Water, and Hygiene				
Preparation of meals				
Kitchen		40 (85)		
Open space		6 (13)		
Room (inside room living in)		1 (2)		
Source of drinking water				
Tube well (hand pump)		3 (6)		
Bore hole (motorized pump)		37 (79)		
Open well (open container with pulley system)		7 (15)		

^ƾ^ Based on World Health Organisation (WHO) criteria [24]. * Matriculated from secondary school, aged 18. ^¶^ Katcha is mud and straw. ^ǂ^ House provided free of charge for brick kiln workers.

**Table 2:** All foods consumed per dietary diversity category.

Food Group Category	All Foods Consumed by Category
1. Grains, white roots and tubers, and plantains	Wheat (flour), maize (flour), potato, rice, turnip, vermicelli
2. Pulses (beans, peas, and lentils)	Lentil, kidney beans, chickpeas, white beans, dried peas
3. Nuts and seeds	Peanuts
4. Dairy	Milk (cow and buffalo), yogurt
5. Meat, poultry, and fish	Chicken, beef, liver, fish
6. Eggs	Chicken eggs
7. Dark green leafy vegetables	Spinach, colocasia leaves
8. Other vitamin A-rich fruits and vegetables	Carrot, pumpkin
9. Other vegetables	Tomatoes, onions, cauliflower, okra, cucumber, radish, apple gourd, aubergine (brinjal), fresh peas, French beans
10. Other fruits	Apple, banana, guava, raisins
A. Other oils and fats	Ghee, oil
B. Sugar-sweetened beverages	Black tea with sugar, green tea with sugar
C. Condiments and seasonings	Chili pepper (red and green), garlic, mixed spices, coriander leaves, coriander seed, salt
D. Other beverages and foods	Black tea, green tea
E. Sweets	Cake, biscuit

**Table 3:** The frequency (%) distribution of the food group diversity score at each time point.

Food Group Score	T1 (n = 47)	T2 (n = 47)	T3 (n = 47)	T4 (n = 46)	T5 (n = 45)
2	4	0	0	4	7
3	17	21	23	17	13
4	53	38	43	37	49
5	17	38	23	37	29
6	9	0	9	4	2
7	0	2	2	0	0

**Table 4:** Baseline blood biomarkers.

Blood Biomarker	n	Mean (SD)	Min	Median	Max	Number below Cutoff or Reference Range, n (%)	Cutoff Value or Reference Range (RR) for Adult Women
Plasma Zinc (µg/L)	47	701.6 (124.2)	435.9	695.6	1060.8	14 (30)	Cutoff: 660 µg/L [30]
Plasma Selenium (µg/L)	47	96.1 (17.1)	56.1	95.3	139.9	8 (17)	Cutoff: 80 µg/L [31]
Plasma Iron (µg/L)	47	962.8 (682.7)	220.6	899.7	3813.7	1 (2)	RR: 280-1620 µg/L [32]
Serum Ferritin (µg/L)	43	55.3 (48.8)	2.94	46.7	302.1	4 (9)	Cutoff: 15 μg/L [27]
Soluble Transferrin Receptor (mg/L)	44	0.76 (0.60)	0.06	0.6	3.01	0	RR: 0.16–4.23 [33]
Hemoglobin (g/L)	45	129 (17)	97	129	213	6 (13)	Cutoff: 120 g/L [28]
Hematocrit (%)	45	39.4 (4.64)	30.1	38.4	62.3	10 (22)	RR: 37–47% [32]
Mean Corpuscular Volume (fL)	45	82.8 (7.4)	57.9	83.5	93.2	5 (11)	RR: 76–100 fL [32]
Mean Corpuscular Hemoglobin Concentration (g/L)	45	325 (18)	268	329	354	12 (27)	RR: 320–360 g/L [34]

## Appendix A

Samples were introduced, via a single line, from an autosampler incorporating an ASXpress™ rapid uptake module (Cetac ASX-520, Teledyne Technologies Inc., Omaha, NE, USA) through a perfluoroalkoxy (PFA) Microflow PFA-ST nebulizer (Thermo Fisher Scientific, Bremen, Germany). All samples and external multi-element calibration standards were diluted as 0.300 mL added to 6 mL of a solution containing (i) 0.5% HNO_3_ (Primar Plus grade), (ii) 2.0% methanol (Fisher Scientific U.K. Ltd., Loughborough, UK), and (iii) three internal standards including ^72^Ge (10 µg/L), ^103^Rh (5 µg L^−1^), and ^193^Ir (5 µg/L) (SPEX Certiprep Inc., Metuchen, NJ, USA). Calibration standards included Cu, Zn, and Se (0, 20, 40, 100 µg L^−1^; Claritas-PPT grade CLMS-2; SPEX Certiprep Inc., Metuchen, NJ, USA). The ICP-MS was operated in “collision–reaction cell mode”, with kinetic energy discrimination, using H_2_ as the cell gas to maximize sensitivity for Se determination and He for all other elements; “in-sample switching” was used to cycle between cell gases with a 30 s refresh period. The quadrupole dwell time was 0.1 s, and 150 scans were used to obtain an average measurement of signal intensity (CPS; counts per second).

The limit of detection (LOD) for all elements was measured as 3 × standard deviation of 10 operational blanks; the limit of quantification (LOQ) was calculated as 10 × standard deviation. Values of LOD and LOQ (µg/L) are shown in Table A1.

**Table A1:** Limits of detection (LOD) and quantification (LOQ) for nine elements; all values are in µg L^−1^.

Element	Cu	Zn	Se
Limit of detection (µg/L)	0.19	1.05	0.020
Limit of quantification (µg/L)	0.64	3.51	0.05

Accuracy was verified by the use of two appropriate reference materials (Seronorm™ L-1 (Lot 1801802) and Seronorm™ L-2 (Lot 1801803); Nycomed Pharma AS, Billingstad, Norway); these were run at the start and the end of the analysis and prepared identically to samples and calibration standards. A total of four individual certified reference material (CRM) analyses were undertaken for both L-1 and L-2. The average recoveries recorded for Seronorm™ L-1 and Seronorm™ L-2 are listed in Table A2.

**Table A2:** Average recovery (%; n = 4) for three elements compared to accredited values.

CRM % Recovery	Cu	Zn	Se
Seronorm L-1	87	100	100
Seronorm L-2	88	104	101

## References

[1] Fanzo J., Hawkes C., Udomkesmalee E., Afshin A., Allemandi L., Assery O., Baker P., Battersby J., Bhutta Z., Chen K. (2019). 2018 Global Nutrition Report.

[2] WHO (2020). Malnutrition. Key Facts. https://www.who.int/news-room/fact-sheets/detail/malnutrition.

[3] UN General Assembly Implementation of the United Nations Decade of Action on Nutrition (2016–2025). https://www.google.com/url?sa=t&rct=j&q=&esrc=s&source=web&cd=&ved=2ahUKEwja3aCBp5PsAhVJURUIHUzRAf0QFjAGegQIAxAC&url=http%3A%2F%2Fwww.fao.org%2F3%2Fa-i6130e.pdf&usg=AOvVaw0sA22shQkFRCcwzqHsGHVt.

[4] Popkin B.M., Corvalan C., Grummer-Strawn L.M. (2020). Dynamics of the double burden of malnutrition and the changing nutrition reality. Lancet.

[5] Harding K.L., Aguayo V.M., Webb P. (2018). Hidden hunger in South Asia: A review of recent trends and persistent challenges. PHN.

[6] Winichagoon P., Margetts B.M. (2017). The Double Burden of Malnutrition in Low-and Middle-Income Countries. https://www.google.com/url?sa=t&rct=j&q=&esrc=s&source=web&cd=&cad=rja&uact=8&ved=2ahUKEwjM3vqTppPsAhVDWxUIHSPsAycQFjABegQIBRAC&url=http%3A%2F%2Fpublications.iarc.fr%2F_publications%2Fmedia%2Fdownload%2F4586%2F53944c7a9c0ec4c1547b35ee5b1f2df6f5c0dc3e.pdf&usg=AOvVaw123QZ4VsnVkY7nUhpFJMgs.

[7] Zia M.H., Ahmed I., Bailey E.H., Lark R.M., Young S.D., Lowe N.M., Joy E.J., Wilson L., Zaman M., Broadley M. (2020). Site-specific factors influence the field performance of a Zn-biofortified wheat variety. Front. Sustain. Food Syst..

[8] Capacci S., Mazzocchi M., Shankar B., Traill B. (2013). The triple burden of malnutrition in Europe and Central Asia: A multivariate analysis. FAO Reg. Off. Eur. Cent. Asia Policy Stud. Rural Transit..

[9] Hawkes C., Ruel M.T., Salm L., Sinclair B., Branca F. (2020). Double-duty actions: Seizing programme and policy opportunities to address malnutrition in all its forms. Lancet.

[10] Government of Pakistan (2019). UNICEF. National Nutrition Survey 2018, Key Findings Report. https://www.google.com/url?sa=t&rct=j&q=&esrc=s&source=web&cd=&cad=rja&uact=8&ved=2ahUKEwiaj5_xqZPsAhXwThUIHWO6D0YQFjAAegQIARAC&url=https%3A%2F%2Fwww.unicef.org%2Fpakistan%2Freports%2Fnational-nutrition-survey-2018-key-findings-report&usg=AOvVaw1-5DhOp8shiA8Xz_ibjJzB.

[11] Bhutta Z.A., Soofi S.B., Zaidi S.S.H., Habib A. (2011). Pakistan National Nutrition Survey. https://www.humanitarianresponse.info/en/operations/pakistan/document/national-nutrition-survey-2011.

[12] Farrell P., Thow A.M., Abimbola S., Faruqui N., Negin J. (2018). How food insecurity could lead to obesity in LMICs: When not enough is too much: A realist review of how food insecurity could lead to obesity in low-and middle-income countries. Health Promot. Int..

[13] Khan G.N., Ariff S., Khan U., Habib A., Umer M., Suhag Z., Hussain I., Bhatti Z., Ullah A., Turab A. (2017). Determinants of infant and young child feeding practices by mothers in two rural districts of Sindh, Pakistan: A cross-sectional survey. Int. Breastfeed. J..

[14] Iqbal S., Zakar R., Zakar M.Z., Fischer F. (2017). Factors associated with infants’ and young children’s (6–23 months) dietary diversity in Pakistan: Evidence from the demographic and health survey 2012–13. Nutrition.

[15] Na M., Aguayo V.M., Arimond M., Stewart C.P. (2017). Risk factors of poor complementary feeding practices in Pakistani children aged 6–23 months: A multilevel analysis of the Demographic and Health Survey 2012–2013. Matern. Child Nutr..

[16] Ali F., Thaver I., Khan S.A. (2014). Assessment of dietary diversity and nutritional status of pregnant women in Islamabad, Pakistan. JAMC.

[17] Lander R.L., Hambidge K.M., Westcott J.E., Tejeda G., Diba T.S., Mastiholi S.C., Khan U.S., Garcés A., Figueroa L., Tshefu A. (2019). Pregnant women in four low-middle income countries have a high prevalence of inadequate dietary intakes that are improved by dietary diversity. Nutrients.

[18] Qureshi Z., Khan R. (2015). Dietary intake trends among pregnant women in rural area of rawalpindi, Pakistan. JAMC.

[19] Das J.K., Achakzai A.B.K., Bhutta Z.A. (2016). Stop stunting: Pakistan perspective on how this could be realized. Matern. Child Nutr..

[20] Victora C.G., Adair L., Fall C., Hallal P.C., Martorell R., Richter L., Sachdev H.S., Maternal and Child Undernutrition Study Group (2008). Maternal and child undernutrition: Consequences for adult health and human capital. Lancet.

[21] Lowe N.M., Khan M.J., Broadley M.R., Zia M.H., McArdle H.J., Joy E.J., Ohly H., Shahzad B., Ullah U., Kabana G. (2018). Examining the effectiveness of consuming flour made from agronomically biofortified wheat (Zincol-2016/NR-421) for improving Zn status in women in a low-resource setting in Pakistan: Study protocol for a randomised, double-blind, controlled cross-over trial (BiZiFED). BMJ Open.

[22] Bingley H., Lowe N., Mehdi R., Haq Z.U., Zaman M. (2018). Developing health service delivery in a poor and marginalised community in North West Pakistan. Pak. J. Med. Sci..

[23] Khadivzadeh T. (2002). Mid upper arm and calf circumferences as indicators of nutritional status in women of reproductive age. EMHJ.

[24] WHO, Expert Consultation (2004). Appropriate body-mass index for Asian populations and its implications for policy and intervention strategies. Lancet.

[25] FAO, FHI 360 (2016). Minimum Dietary Diversity for Women: A Guide for Measurement.

[26] FAO (2018). Dietary Assessment, A Resource Guide to Method Selection and Application in Low Resource Settings.

[27] WHO (2011). Serum Ferritin Concentrations for the Assessment of Iron Status and Iron Deficiency in Populations.

[28] WHO (2011). Haemoglobin Concentrations for the Diagnosis of Anaemia and Assessment of Severity.

[29] UN, WHO (2001). Iron Deficiency Anaemia: Assesment, Prevention and Control: A Guide for Programme Managers.

[30] Brown K.H., Rivera J.A., Bhutta Z., Gibson R.S., King J.C., Lönnerdal B., Ruel M.T., Sandtröm B., Wasantwisut E., Hotz C. (2004). International Zinc Nutrition Consultative Group (IZiNCG) technical document# 1. Assessment of the risk of zinc deficiency in populations and options for its control. Food Nutr. Bull..

[31] Thomson C. (2004). Assessment of requirements for selenium and adequacy of selenium status: A review. Eur. J. Clin. Nutr..

[32] Royal College of Physicians and Surgeons of Canada (2017). Clinical Laboratory Tests—Reference Values. https://www.google.com/url?sa=t&rct=j&q=&esrc=s&source=web&cd=&ved=2ahUKEwjp_o-bwI7sAhUdUBUIHR23DCAQFjADegQIARAB&url=http%3A%2F%2Fwww.royalcollege.ca%2Frcsite%2Fdocuments%2Fcredential-exams%2Fclinical-lab-tests-reference-values-e.pdf&usg=AOvVaw0esrInJySYgpP5_Tg2umOV.

[33] Suega K., Kandarini Y., Tubung J. (2019). Role of Soluble Transferrin Receptor and Transferrin Receptor-Ferritin Index to Detect Iron Deficiency Anemia in Regular Hemodialysis Patients. OAMJMS.

[34] The Royal Wolverhampton NHS Trust (2017). Haematology Normal Adult Reference Ranges. https://www.royalwolverhampton.nhs.uk/services/service-directory-a-z/pathology-services/departments/haematology/haematology-normal-adult-reference-ranges/.

[35] Harding K.L., Aguayo V.M., Namirembe G., Webb P. (2018). Determinants of anemia among women and children in Nepal and Pakistan: An analysis of recent national survey data. Matern. Child Nutr..

[36] Hunt J.R. (2003). Bioavailability of iron, zinc, and other trace minerals from vegetarian diets. AJCN.

[37] Tavajjoh M., Yasrebi J., Karimian N., Olama V. (2011). Phytic Acid Concentration and Phytic Acid: Zinc Molar Ratio in Wheat Cultivars and Bread Flours, Fars Province, Iran. J. Agric. Sci. Technol..

[38] Delimont N.M., Haub M.D., Lindshield B.L. (2017). The impact of tannin consumption on iron bioavailability and status: A narrative review. Curr. Dev. Nutr..

[39] Disler P., Lynch S., Torrance J., Sayers M., Bothwell T., Charlton R. (1975). The mechanism of the inhibition of iron absorption by tea. S. Afr. J. Med..

[40] Geerligs P.P., Brabin B., Mkumbwa A., Broadhead R., Cuevas L.E. (2003). The effect on haemoglobin of the use of iron cooking pots in rural Malawian households in an area with high malaria prevalence: A randomized trial. Trop. Med. Int. Health.

[41] Charles C.V., Summerlee A.J., Dewey C.E. (2011). Iron content of Cambodian foods when prepared in cooking pots containing an iron ingot. Trop. Med. Int. Health.

[42] Tripp K., MacKeith N., Woodruff B.A., Talley L., Mselle L., Mirghani Z., Abdalla F., Bhatia R., Seal A.J. (2010). Acceptability and use of iron and iron-alloy cooking pots: Implications for anaemia control programmes. Public Health Nutr..

[43] Alam A., Ali A., Lodhi A., Alam S., Rauf N. (2016). Serum selenium concentration and subsequent risk of diabetes miletus in Pakistan. JDCM.

[44] Pan American Health Organization, UNICEF (2013). ProPAN: Process for the Promotion of Child Feeding: A Tool to Improve Infant and Young Child Feeding.

